# Malignant nodular melanoma of the vulva: a rare and aggressive tumour of the female genital tract (case report)

**DOI:** 10.11604/pamj.2021.38.115.25864

**Published:** 2021-02-03

**Authors:** Moshawa Calvin Khaba, Matsiane Luciah Lekala, Setheme Daniel Mosehle

**Affiliations:** 1 Department of Anatomical Pathology, National Health Laboratory Services, Sefako Makgatho Health Sciences University, Pretoria North, Gauteng Province, South Africa,; 2 Department of Obstetrics and Gynaecology, Dr George Mukhari Academic Hospital, Sefako Makgatho Health Sciences University, Pretoria North, Gauteng Province, South Africa

**Keywords:** HIV, malignant, nodular melanoma, metastasis, case report

## Abstract

Malignant melanoma of the vulva is a rare and aggressive tumour with dismal prognosis. It tends to recur and metastasize early. Surgical excision with or without regional lymph node dissection is still the treatment of choice with adjuvant therapy decided on a case by case. Furthermore, HIV infection has been associated with more aggressive disease. Herein we present a 45-year-old HIV-infected female patient on antiretroviral therapy who presented with vulval ulcer for one year. On examination, she had ulcerated nodule on the labia majora. Radiology showed vulvovaginal tumour without involvement of the adjacent organs. Malignant melanoma was confirmed on both the incisional biopsy and vulvectomy. She responded poorly to radiotherapy. Furthermore, she presented with recurrence and metastatic disease a month after surgery. She was lost to follow-up clinic.

## Introduction

Malignant melanoma originating from the female genital tract is rare [1]. It arises from the vagina, cervix and vulvar, and accounts for 1-3% of all melanoma diagnosed in women [1, 2]. Approximately 5% of melanomas diagnosed in women are from the vulvar and constitute 8-10% of all malignant tumours of the vulva [1, 3]. Although they are extremely rare in the vagina and the cervix, it is the second most common malignancy of the vulva [4]. They occur mainly in the clitoris and labia minora or labia majora [5]. The prognosis is usually poor with unpredictable clinical outcome and a 5-year survival rate. People infected by human immunodeficiency virus (HIV) have a higher risk of malignancies, and melanoma is not an exception. The incidence of melanoma is increased in HIV-infected people compared to non-infected; and it is associated with more aggressive behaviour [5]. Surgical excision with regional lymph node dissection remains the treatment of choice [2]. This manuscript describes a rare vulvar melanoma in an HIV-infected patient with aggressive behaviour.

## Patient and observation

**Figure 1:**
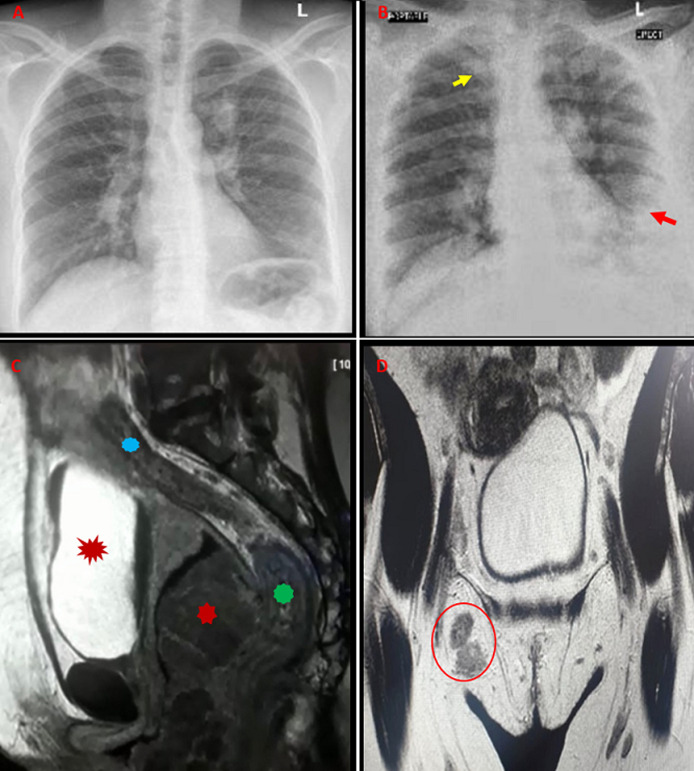
A) initial normal appearing CXR; B) metastasis evidenced by upper lobes nodular infiltrate and pleural effusion; this CXR was performed after 1 month of surgery; C) pelvic MRI reveals an ill-defined vulvovaginal tumour not involving the bladder, uterus or rectum; D) CT scan show right inguinal lymphadenopathy

A 45-year-old female patient who presented with a vulvar ulcer for one year without other symptoms. She was HIV infected with CD4+ of 291 cells/μL on highly active retroviral treatment. She was previously treated for pulmonary tuberculosis for six months. She had no other co-morbidities or family history of malignancy. On examination, she had good performance score. The abdomen was soft with right inguinal lymphadenopathy. Per vaginal examination revealed an ulcerated nodule on the right labia majora extending from the right labia minora into the right lower 2/3 of the vagina. This lesion was close to the anal sphincter, but did not involve the pelvic bone. It was draining foul smelling dark discharge. She did not have pigmented lesion/nevus on any part of the body. A biopsy of the ulcer showed malignant melanoma. The patient was counselled about the disease and the prognosis thereof. She was further investigated for extent of the disease and possible metastasis. The chest X-ray (CXR) was unremarkable (Figure 1A). The Magnetic Resonance Imaging (MRI) revealed a vulvovaginal tumour which pushed the uterus superiorly. It did not show bladder or rectal involvement (Figure 1C). Computed Tomography (CT) showed pelvic lymph nodes metastasis (Figure 1D). The full blood count showed hemoglobin of 9.2 g/dl while the rest of the blood investigation was unremarkable. She was offered pelvic exenteration but declined. A hemivulvectomy and vaginectomy with inguinofemoral and pelvic lymphadenectomy was performed.

**Figure 2:**
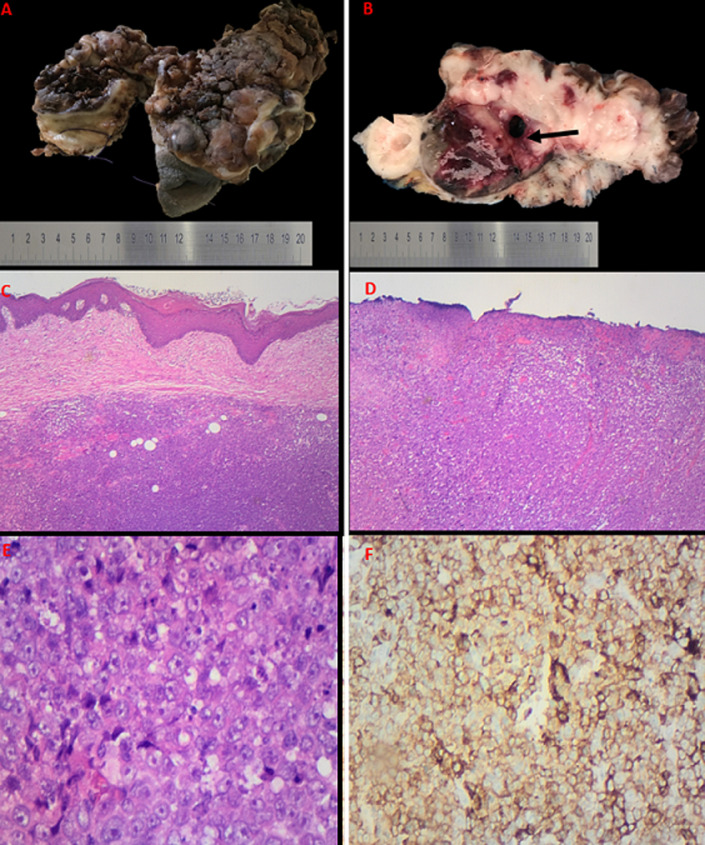
A) ulcerated and multinodular tumour with focal labia majora; B) cut section show nodular, white-tan and hemorrhagic tumour; C) uninvolved epithelium; D) ulcerated epithelium with underlying nodular tumour; E) large, atypical cells with vesicular nuclei and prominent inclusion-like nucleoli; F) immunohistochemistry show tumour cells immunoreactive for Melan A

An ulcerated, multinodular and rubbery tumour measuring 90x50x25mm was received (Figure 2A, Figure 2B) with pelvic and inguinal lymph nodes for histopathological assessment. Microscopy showed a malignant nodular melanoma evidenced by an ulcerated tumour arranged in a nodular pattern without epithelial component (Figure 2C, Figure 2D). The tumour cells had pleomorphic and vesicular nuclei with prominent inclusion-like nucleoli. Intracytoplasmic melanin pigment was not seen (Figure 2E). The tumour corresponded to Clark level V and Breslow stage V. There was no brisk intratumoural lymphocytic infiltrate. The mitotic count was 15 per square millimeter. The tumour cells were immunoreactive for melanin A (Figure 2F), HMB45 and S-100 protein. Molecular studies confirmed a BRAF pV600E mutation. The tumour was present at the vaginal resection margin and 1mm from the closest soft tissue excision margins. Only 3 of the 17 inguinofemoral lymph nodes showed metastatic diseases.

Post-surgically, she presented with recto-vaginal fistula after 23 days. A diverting colostomy was performed and discharged to home. She came back after a month to the gynaecology clinic with shortness of breath, weakness and pain of the lower limbs. Per vaginal examination revealed bleeding and non-healing operation side and a well colostomy. Although the biopsy was not done at this stage, a clinical diagnosis of local recurrence was made. A CXR at this point showed upper lobes nodules and left pleural effusion in keeping with lung metastasis (Figure 1B). She was referred to radiation oncology where she received palliation radiotherapy of 8 Grays and one fraction of radiation. She was discharged from oncology due to poor treatment response. She was eventually discharged home for palliation care and has since been lost to follow-up clinic.

## Discussion

In general, melanomas are some of the most aggressive cancers in human, despite their location [3]. Vulvar melanomas are usually diagnosed late or at advanced stage due its location and absence of early presenting clinical features [6]. Late presenting features include pigmented, painful and bleeding lesion which can be ulcerated at times. Patients may also complain about pruritus and discomfort [3, 7, 8]. One-third of the patients may present with lymph nodal involvement which is attributed to the rich vascular and lymphatic network of the vulva which also contributes to the early development of metastasis. They tend to have a local recurrence and the common site being the groin while the lung is the most common site of metastasis followed by the liver, and the brain [6]. The index patient presented late with ulcerated nodule and regional lymphadenopathy suggestive of metastatic disease which was confirmed histologically. The common histological variant is lentiginous melanoma, followed by superficial spreading melanoma and nodular melanoma; and most of the tumours are amelanotic [7, 8].

Histologically, the common cell types are epithelioid, followed by spindle and mixed type. Confirmatory immunohistochemical stains include positive S-100 protein, melan A and HMB-45 [9]. Carcinomas of the vulva and clear cell sarcoma of soft tissue are amongst the differential diagnosis [8]. Exploring genetic alterations and pathways associated with this disease may help to move toward more specific therapeutic interventions in these patients [4]. The two common mutations in malignant melanomas are *NRAS* and *BRAF* point mutations. They occur early and remain throughout the course of disease including metastasis [4, 9]. They can be considered potential treatment target; hence it is advised that all female genital tract melanoma undergo molecular studies. Confirmed *BRAF* mutation in this case was also used to exclude clear cell sarcoma of soft part which was a morphological differential diagnosis. BRAF mutation is also associated with more aggressive behaviour. Whilst surgery still remains the treatment of choice, radical surgery has not proven to increase patient´s survival [8]. Wide local excision with a 1cm surgical margin is recommended for lesions with a depth of less than 1mm and *en bloc* resection for deeper lesions, with a safety margin of 2-3cm and regional lymphadenectomy [1, 7-9].

There are no differences in recurrence or survival between women treated with excision or hemivulvectomy compared to those who underwent radical vulvectomy and lymphadenectomy [6]. Current National Comprehensive Cancer Network Guidelines recommend that primary surgical excision aim for surgical margins of 0.5-1.0cm for *in-situ* melanoma, a 1cm margin for melanomas with thickness less than 1mm, 1-2cm margins for a thickness of 1-2mm, and 2cm margins for melanomas with thickness greater than 2.01mm. Adjuvant therapy can be directed by lymph node status [1]. With regards to the index patient, clear surgical margins were attempted; however, there was positive histological vaginal excision margin with associated regional lymph node metastasis. The difficulty in obtaining clear surgical margin is usually attributed to the anatomical position of this tumour. There is still no consensus on the preferred stage to use between International Federation of Gynecology and Obstetrics (FIGO) and American Joint Committee on Cancer´s (AJCC) staging methods [7, 9].

The FIGO staging system seemed to be a suboptimal predictor of recurrence and survival while the AJCC staging was found to be more appropriate to determine prognosis and therapy in these patients [7]. The Society for Immunotherapy of Cancer (SIC) recommends adjuvant therapy for patients at increased risk for recurrence [1]. Prognosis is determined by tumour site, depth of invasion, ulceration, satellite lesions, local extension to the urethra or vagina and lymph node mestastasis [6, 10]. The literature demonstrates that 5-year survival rates are around 20-56%, irrespective of stage [10]. The index patient had poor prognostic features which included ulceration, Breslow and Clark level V, positive vaginal excision margins and lymph node metastasis. The patients responded poorly to radiotherapy and later developed recurrent disease; both local and distant. In view of the accelerated course of the disease since the histopathological confirmation and HIV status of the patient, this further confirms the more aggressive nature of this disease in HIV infected patients compared to non-infected patients. It is uncertain if the patient is still alive, as she had been lost to follow-up visit.

## Conclusion

The accurate and early diagnosis is important in vulvovaginal melanoma because of its unfavourable and unpredictable prognosis; with the tendency to recur and metastasize. HIV infection is associated with more aggressive behaviour and poorer prognosis compared to non-infected patients. Surgical treatment constitutes the most fundamental therapeutic management as adjuvant treatment has not shown to improve prognosis/survival. Molecular investigation has a role to play in management of this tumour as mutation of certain genes may have a role in targeted therapy to improve survival and outcome.
